# Molecular Mechanisms and Pathways as Targets for Cancer Prevention and Progression with Dietary Compounds

**DOI:** 10.3390/ijms18102050

**Published:** 2017-09-25

**Authors:** Nagisa Nosrati, Marica Bakovic, Gopinadhan Paliyath

**Affiliations:** 1 Department of Plant Agriculture, University of Guelph, Guelph, ON N1G 2W1, Canada; nnosrati@uoguelph.ca; 2 Department of Human Health and Nutritional Sciences, University of Guelph, Guelph, ON N1G 2W1, Canada; mbakovic@uoguelph.ca

**Keywords:** bioactive food ingredients, inflammatory pathways, cancer, antioxidants, inflammation, cell cycle, cell migration, epigenetic modifications, microRNA, inflammatory bowel diseases (IBD)

## Abstract

A unique feature of bioactive food ingredients is their broad antioxidant function. Antioxidants having a wide spectrum of chemical structure and activity beyond basic nutrition; display different health benefits by the prevention and progression of chronic diseases. Functional food components are capable of enhancing the natural antioxidant defense system by scavenging reactive oxygen and nitrogen species, protecting and repairing DNA damage, as well as modulating the signal transduction pathways and gene expression. Major pathways affected by bioactive food ingredients include the pro-inflammatory pathways regulated by nuclear factor kappa B (NF-κB), as well as those associated with cytokines and chemokines. The present review summarizes the importance of plant bioactives and their roles in the regulation of inflammatory pathways. Bioactives influence several physiological processes such as gene expression, cell cycle regulation, cell proliferation, cell migration, etc., resulting in cancer prevention. Cancer initiation is associated with changes in metabolic pathways such as glucose metabolism, and the effect of bioactives in normalizing this process has been provided. Initiation and progression of inflammatory bowel diseases (IBD) which increase the chances of developing of colorectal cancers can be downregulated by plant bioactives. Several aspects of the potential roles of microRNAs and epigenetic modifications in the development of cancers have also been presented.

## 1. Introduction

According to World Health Organization, cancer is the second cause of death globally after cardiovascular diseases. An estimated 8.2 million people die from cancer each year, representing 13% of all deaths worldwide [1]. Cancer results from uncontrolled rapid division of malignant cells that grow beyond their usual boundaries. Unlike normal cells, cancerous cells do not respond to the controlling signals; consequently, they grow and divide in an uncontrolled manner, infecting normal tissues and organs, and in some cases, ultimately spreading throughout the body. This feature is reflected in several aspects of cell behavior that distinguish cancer cells from their normal counterparts [2].

There are over 100 different types of cancers [3]. The type of the cell that tumors originate from classifies cancers. Carcinomas, cancers derived from epithelial cells of breast, prostate, lung, pancreas and colon, cause ~90% of all human deaths from cancer; lymphomas are cancers of the immune organs such as spleen, white blood cells and lymph glands; leukemias are cancers of blood forming bone marrow; sarcomas are cancers of fibrous connective tissues of bone, cartilage, fat tissue, muscle and neurons; and germ cell tumors are derived from pluripotent stem cells presented in the testicles and ovary [4].

Early detection and effective treatment help increase survival rates of cancer patients. Therefore, comprehensive plans are needed to improve prevention and treatment of cancer. Evidence from epidemiological and experimental studies proved that high intake of fruits and vegetables decreases chronic degenerative diseases, and importance of consuming a balanced diet in relation to cancer prevention has received particular interest. Fruits and vegetables are rich sources of different classes of bioactive molecules. Dietary polyphenols, the most studied group, play important roles in preventing and managing cancer due to their antioxidant and anti-inflammatory activities. Several pieces of evidence have accumulated for cancer prevention by bioactives; especially, bioactives such as phytoestrogens from soy and flaxseed oil, phenolics from olives and olive oil, resveratrol from nuts and red wine, lycopene from tomatoes, organosulfur compounds from garlic and onion, isothiocyanates in cruciferous vegetables, and monoterpenes in citrus fruits and herbs [5,6,7]. Many bioactive phytochemicals isolated from garlic, turmeric and green tea [8,9] are being tested in human cancer clinical trials.

Phytochemicals possess various types of activities. They can detoxify free radicals, alter the expression of genes involved in metabolism, cell survival, proliferation and antioxidant defense; protect and repair DNA damage, and cause cell cycle arrest and apoptosis [10,11,12]. Because of their complex chemical structures, bioactives can act at multiple sites in the cancer development resulting in prevention and progression of cancer [13,14,15]. Bioactive compounds that are involved in cancer prevention act by regulating the expression and activity of transcription factors, growth factors, inflammatory mediators, and cell cycle intermediates. Bioactive ingredients with therapeutic properties show inhibition of cancer progression by suppressing cell survival, proliferation, cell invasion, angiogenesis and metastasis. Epigenetic alterations accumulated over time can be involved in the pathogenesis of cancer. Bioactive-dependent epigenetic variations induce the effects on genome stability, mRNA and protein expression, and can cause multiple metabolic changes [16]. The overall goal of the present review is to elaborate the major steps involved in defining the effects of functional food ingredients on cancer, and discuss the main molecular mechanisms behind these processes.

## 2. Inflammation, Cancer, and Regulation by Dietary Intakes

Inflammation, a major link between risk factors and cancer, is being identified as the commencing point for several forms of cancer [17]. Diet and life style are known risk factors, such as obesity, environmental pollutants, alcohol, smoking, irradiation, high fat diet, etc. Acute inflammation induced by pathogen attack persists for a short period, while chronic inflammation predisposes the body to develop cancer. Activation of two major inflammatory pathways, NF-κB and Signal transducer and activator of transcription 3 (STAT3), is associated with most cancers [18]. Hypoxia and acidosis observed in solid tumors increase NF-κB. NF-κB and STAT3 regulate gene expression associated with inflammation, cell survival, cell proliferation, invasion, angiogenesis, and metastasis. Suppression of these two pathways results in the suppression of tumor growth, a clear criterion for chemo preventive agents. Colonic mucosal biopsies from patients either suffering from ulcerative colitis or Crohn’s disease have increased levels of inflammation marker compounds and decreased levels of anti-oxidant enzymes, suggesting the role of increased oxidative stress and decreased antioxidant defenses as well as NF-κB activation in the development of colorectal cancer [19,20].

Dietary components affect genomic and non-genomic processes that could both promote beneficial, disease-preventing processes and inhibit overactive, cancer promoting processes. Multiple studies using cell lines, animal models, and human clinical trials consistently showed the ability of bioactives in causing cytotoxicity to cancer cells [21]. Research showed that grape and wine polyphenols exhibit selective cytotoxicity to the breast cancer cells (MCF-7) by comparison to the normal mammary epithelial cells (MCF-10A). Polyphenols triggered necrotic cell death of cancer cells without any deleterious effect on noncancerous cells. In response to polyphenols the MCF-7 cells showed an increase in cytoplasmic calcium level and arrest at G1/S and G2/M phase of the cell cycle. In addition, red grape polyphenols are able to inhibit the tumor growth in vivo when the triple negative breast cancer cells MDA-MB-231 were implanted in the athymic nu/nu mice. These results demonstrated that grape polyphenols act at multiple critical control points in the cancer cell biochemical pathways such as the inflammatory pathways, exerting selective cytotoxicity. NF-κB related pathway is one of the main pathways, which can be targeted by polyphenols in grapes and wine, through induction of the phase II enzymes, which was supported by gene expression analysis including *CK2*, *FAS*, *LEF1*, *PRKCE* and *PTGS2* genes [22,23,24,25]. At present, it is not clear as to what makes the cancer cells more susceptible to polyphenols. Understanding these aspects will help develop clear strategies for dietary interventions using functional food products for cancer treatment and prevention.

## 3. Plant Bioactives and Targeting Antioxidant Pathways

**Figure 1:**
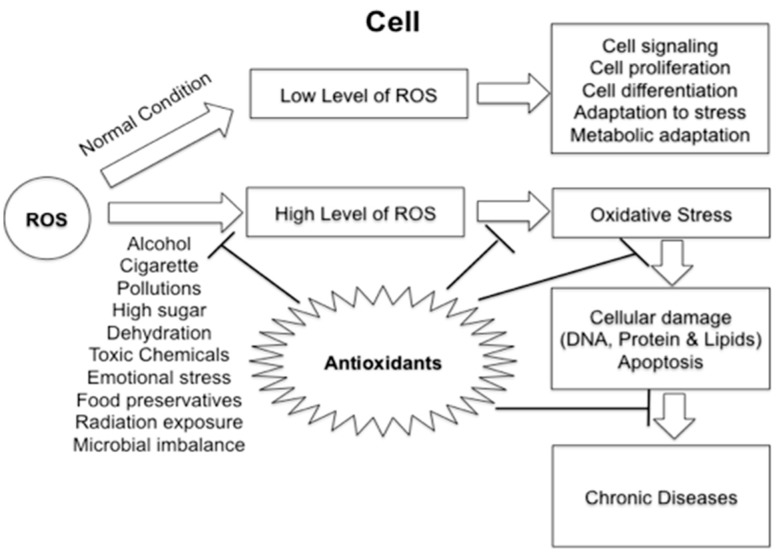
Prevention of oxidative stress by antioxidants. Antioxidants neutralize ROS related pathways. ROS play important roles in cell signaling, cell proliferation, cell differentiation, adaptation to stress and metabolic adaptation. Raised levels of the ROS could lead to cellular damage and chronic disease development. Antioxidants modulate ROS levels and prevent cell damage through various mechanisms.

Oxidative stress is tightly regulated by a balance between production and removal of free radicals, which are formed naturally in the body with important roles in cell signaling. Free radicals can be hazardous to the body and damage all major components of cells, including DNA, proteins and cell membranes. In particular, DNA damage plays a critical role in the development of cancer [26]. According to epidemiological data, some bioactive compounds inhibit different stages of carcinogenesis, including initiation, promotion and cancer progression, by reducing reactive oxygen species (ROS) levels (Figure 1). Fruits, vegetables and grains are rich sources of dietary antioxidants such as vitamin E, vitamin C, polyphenols (flavonoids such as quercetin, catechin, naringenin and anthocyanins that include sugar derivatives of cyanidin, pelargonidin, petunidin, peonidin and malvidin), carotenoids and essential minerals such as Selenium and Zinc (that act as cofactors for essential host pathway enzymes [27]. Consumption of diets high in vitamin E, vitamin C and β-carotene has been shown to reduce cervical, stomach and lung cancers, respectively [28,29,30]. Anti-cancer effects of strawberry flavonoids include scavenging of ROS, reducing DNA damage and decreasing cancer cell proliferation [31].

The human body is increasingly absorbing xenobiotics including drugs, environmental pollutants, food-derived preservatives and hormones. These components can be mutagenic and carcinogenic, especially during the detoxification process, when they are made soluble. The phytochemicals are able to prevent the initiation of carcinogenesis by inducing xenobiotic-detoxifying enzymes. Phase I and II enzymes such as glutathione *S*-transferase (GST) and UDP-glucuronyl transferase (UDP-GT) are responsible for metabolism of various endogenous or exogenous substrates to protect the cells from cellular damage, arising from the activation of carcinogenic factors [32].

The main regulator of phase II enzymes is nuclear factor F-related factor 2 (Nrf2). Sulforaphane activates Nrf2 localized in the cytoplasm and bound to Kelch-like ECH-associated protein 1 (Keap1), which limits Nrf2 activity by retaining it in the cytoplasm and increasing its proteasomal degradation. In response to oxidative stress, Nrf2 dissociates from Keap1, translocates to the nucleus, and binds to the antioxidant responsive element (ARE) promoting expression of antioxidant enzymes [33]. Oral administration of sulforaphane activates Nrf2 pathway and significantly reduces tumor size and increases apoptosis through activation of caspase 3 and cytochrome c release in bladder cancer cells [34]. Sulforaphane also induces antioxidative and anti-proliferative effects on human bronchial epithelial cells via ROS-mediated mechanism, and activation of PI3K and MEK/Erk1/2 signaling pathways. This resulted in up-regulation of intracellular oxidants, Erk1/2 phosphorylation, and nuclear accumulation of Nrf2, all of which increased in HO-1 expression and a decrease in cell growth [35].

Polyphenols in the diet play their antioxidant role in multiple ways to scavenge cancer initiating free radicals, activation of transcription of cytoprotective enzymes involved in detoxification of xenobiotics, and modulation of signal transduction systems [36]. Antioxidants can induce the Keap1/Nrf2/ARE (Kelch ECH associating protein 1/NF-E2-related factor 2/Antioxidant Response Elements) pathway resulting in increased expression of phase 2 detoxification enzymes and antioxidant enzymes [37,38]. Polyphenols including flavonoids, anthocyanins, etc. that contain ortho-dihydroxy groups have been found to stimulate the transcription of genes encoding antioxidant enzymes through the Keap1/Nrf2/ARE pathway and thereby enhance detoxification. Glucoraphanin in broccoli gets metabolized to sulforaphane, which acts through the KEAP1-Nrf2 pathway. Epidemiological studies have shown strong inverse associations between crucifer vegetable intake and the incidences of cancers affecting lung, pancreas, bladder, prostate, thyroid, skin, stomach and colon [39]. Sulforaphane caused cytotoxicity and G2/M arrest in HT-29 colon cancer cells and MCF-7 breast cancer cells [40]. The induction of apoptosis in cancer cells by sulforaphane involved the activation of Bcl2 proteins Bax and Bak [41]. Sulforaphane causes inhibition of tubulin polymerization, activation of G2/M kinases and histone deacetylation resulting in cell cycle arrest and apoptosis. These mechanisms may enable sulforaphane to inhibit carcinogenesis even after initiation [42]. Selenium in broccoli has also been found to have cancer-preventive effects through its function as an activator of antioxidant and detoxification systems [43]. Expression levels of antioxidant enzymes are reduced in cancer cells [44]. In several human trials, fruit polyphenols have been found to down regulate pro-inflammatory cytokines and chemokines [45,46,47]. Thus, dietary antioxidants are potent candidates for use as bioactives to enhance the function of the antioxidant defense system during normal living conditions thus preventing inflammation and decreasing the chance of developing cancer [48,49].

*Chrysanthemum zawadskii* and licorice *Glycyrrhiza uralensis* extracts are well known for their therapeutic aspects of inflammatory diseases [50,51,52]. The extracts also have the potential to induce Nrf2, which has an important role in defense against acute inflammation. Chemopreventive agents, Isoliquiritigenin isolated from the roots of *Glycyrrhiza uralensis* and some isothiocyanate analogs can inhibit NF-κB via the down-regulation of IKK, ERK1/2 and p38 phosphorylation, consequently suppressing the pro-inflammatory mediators such as TNF-α, COX-2, IL-6, iNOS and IL-1β [53,54,55,56].

Among spices, curcumin and its analogs have attracted great attention as cancer-preventive agents through their anti-cancer activities including inhibition of cell proliferation, anti-invasive activity, and inhibition of angiogenesis [57,58,59]. These components were effective on three colorectal cell lines, SW480, HT-29 and HCT116. The molecular targets of inhibition by curcumin include critical control points in the signal transduction pathways such as NF-κB, COX-2, 5-LOX involved in prostaglandin biosynthesis pathway, receptors such as EGFR, HER2, apoptosis regulators such as Bcl-2 and Bcl-XL, caspases, and kinases such as Akt, MAPK, as well as transcription activators and factors such as AP-1 and STAT3. Curcumin, another natural Nrf2 activator, inhibits mouse liver lymphoma through activating Nrf2 enzymes, promoting tumor suppressor p53 and reducing TGF-β and COX2 [60]. Curcumin, through a ROS-dependent mechanism, is also able to induce activation of caspase 8, 2 and 9, alteration of mitochondrial membrane potential, release of cytochrome *c*, activation of caspase-3 and concomitant PARP cleavage, and apoptosis in HUT-78 lymphoma cells [61].

Indole 3-carbinol (I3C) is another bioactive component of crucifer vegetables, with actions similar to that of sulforaphane. I3C is also enriched in broccoli. Several earlier studies have shown the beneficial effects of I3C in breast cancer prevention. Hormone dependent cancers such as breast cancer are promoted by hormone analogs with activity higher than that of natural estrogen. Estrogen is normally metabolized and eliminated from the body through the phase 1/phase 2 detoxification system. This process involves the hydroxylation of estrogen at 2 C or 16 C position on the ring. Thus a higher ratio of 2-hydroxy estrone to 16-hydroxy estrone may determine the cancer preventive status. Consumption of 300 mg of I3C per day caused an increase in the ratio of 2 α-hydroxy estrone to 16 α-hydroxy estrone in woman who are at a risk of developing breast cancer [62]. Metabolism of estrogen favouring 2-hydroxy estrone is influenced by race, ethnicity, and dietary factors (increased consumption of fiber, polyphenols, crucifer vegetables, fruits) [63]. Phosphorylation and activation of estrogen receptor by estrogen was inhibited by I3C [64]. I3C has been found to inhibit prostate cancer [65]. A reduction in respiratory papilloma was observed in response to I3C intake in 66% of the patients [66].

## 4. Arrest of Cancer Cell Cycle by Bioactive Compounds

Berries induce apoptosis through cell-cycle arrest at G1 phase via induction of WAF1/p21 and inhibition of cdk4, cdk6, cyclin D1 and cyclin D3 [67]. They suppress tumor necrosis factor α (TNF-α) induced COX-2 expression followed by down-regulation of activator protein-1 (Ap-1) and NF-κB [67,68]. They are also able to inhibit Wnt signaling and angiogenesis [69].

Citrus flavonoids and limonoids arrest the cell cycle at S and G2/M phases [70]. Limonexic acid and β-sitosterol glucoside block the cell cycle in G2/M phase, induce cytotoxicity and cause apoptosis [71,72].

Genistein, quercetin, daidzein, luteolin, kaempferol, apigenin and epigallocatechin, all are capable of blocking the cell cycle by modifying the activity of the cyclin-dependent kinases (CDKs) [32]. Minute structural differences can alter the mode of action of bioactives. Quercetin, luteolin and daidzein are able to block the cells at G1 phase by inhibiting the activity of CDK2, while kaempferol, apigenin and genistein arrest the cell cycle at G2 phase by blocking CDK1 through inducing CDK inhibitors such as p21 and p27 [73,74]. Certain flavonoids such as tangeretin inhibit hepatic cancer in initiation and progression stages [75]. Luteolin and apigenin also prevent liver cancer development by inhibiting CDKs [76,77].

Phenethyl isothiocyanate (PEITC) from cruciferous vegetables such as broccoli and cabbage causes G2/M cell cycle arrest and apoptosis of myeloma cells [78]. PEITC induces apoptosis in metastatic lung cancer cells via caspase-3 activation and cell cycle arrest at the G2/M phase by modulation of cyclin B1 expression [79]. Curcumin, a component of turmeric, derived from the rhizomes of *Curcuma longa* inhibits proliferation of human pancreatic cancer cells by activation of Ataxia telangiectasia mutated (ATM)/checkpoint kinase 1 (ChK1)/Cdc25C, blocking cyclin B1/Cdk1 activity and arresting cells at G2/M check point [80,81].

## 5. Inhibition of Cancer Cell Proliferation and Migration by Plant Bioactives

Combating cancer requires bioactive components with potential to target multiple signaling pathways. Isoflavones can prevent carcinogenesis through inhibiting cell proliferation and inducing apoptosis. Isoflavones can affect multiple cell signaling pathways important for cancer growth such as NF-κB, Akt, MAPK, Wnt, Notch, p53 and androgen receptor (AR) signaling pathways leading to apoptosis [82,83,84,85,86]. Isoflavones can induce apoptotic cell death, either alone, or in a combination with conventional therapies (chemotherapy and radiotherapy). Honokiol, a bioactive isoflavone from *Magnolia*, inhibits lung cancer cell migration (A549, H1299, H460 and H226 NSCLC). Honokiol suppresses PGE2-mediated migration of NSCLC lung cancer cells by induction of COX-2 and inhibition of NF-κB. PGE2 regulates β-catenin signaling, which contributes to cancer cell migration. Treatments of lung cancer cells with honokiol resulted in degradation of cytosolic β-catenin, reduced the nuclear accumulation of β-catenin, and expression of matrix metalloproteinases (MMP-2 and MMP-9). MMPs are down-stream targets of β-catenin and play a crucial role in metastasis. Honokiol enhanced the levels of casein kinase-1a (CK1α), glycogen synthase kinase-3 β (SK3 β); and β-catenin phosphorylation at critical residues Ser45, Ser33/37 and Thr41. These events are important for degradation and inactivation of β-catenin [87,88]. The same mechanism was established for genistein, an important soybean isoflavone, which induced cytotoxicity in prostate cancer cells. Genistein up-regulated the expression of GSK-3β, which phosphorylates β-catenin leading to its degradation and the inactivation of Wnt/β-catenin signaling, cell growth and migration [89,90,91].

## 6. Deregulation of Hypoxia and Glucose Metabolism in Cancer

One of the important features of cancer cells is elevated glucose consumption and its catabolism by glycolysis, causing an accumulation of lactate. Lactate dehydrogenase A (LDH-A) is an enzyme which uses lactate for energy production and NAD^+^ regeneration, which is a novel therapeutic target for cancer [92]. LDH-A is over-expressed in various types of cancer including renal, breast, gastric and nasopharyngeal cancer. Cancer glycolysis is regulated by hypoxia inducible factor (HIF) and LDH-A is a known target of HIF-1a. HIF activation increases expression of the genes for glucose transport and metabolism, as well as lactate formation and export from the cells. Furthermore, the activity of pyruvate dehydrogenase complex (PDH), an important enzyme in glucose metabolism, is reduced by HIF [93,94].

Chinese herbal medicine, *Spatholobus suberectus* is a compelling LDH-A inhibitor. It induces cell cycle arrest and anti-LDH-A activity in breast cancer estrogen-dependent (MCF-7) cells and estrogen-independent (MDA-MB-231) cells. Epigallocatechin also inhibited LDH-A activity and caused cell apoptosis. LDH-A is regulated by HIF-1a and epigallocatechin caused dissociation of Hsp90 from HIF-1a and subsequent HIF-1a degradation. Epigallocatechin also inhibited breast cancer cell growth in vivo, HIF-1a- and LDH-A- expression and triggered apoptosis without significant toxic side effects. Epigallocatechin can be considered as a pharmacologically effective compound to inhibit HIF-1a and LDH-A in cancer cells [95].

Curcumin caused LDH-A release from mitochondria, by modifying mitochondrial membrane potential, procaspase-3 and -9 cleavage, as well as apoptosis, in a dose- and time-dependent manner. It resulted in cell cycle arrest in S phase, accompanied by the release of cytochrome c, a significant increase of Bax and p53 levels, and a marked reduction of Bcl-2 and survivin in human colorectal carcinoma cells [96,97].

## 7. Inflammatory Bowel Diseases and down Regulation of Immune System by Dietary Components

Inflammatory bowel diseases (IBD) are a group of immune-mediated intestinal inflammatory diseases induced by environmental stimulation and genetic susceptibility [98,99]. IBD includes two main types of diseases, Crohn’s disease (CD) and ulcerative colitis (UC). At present, the etiology and mechanisms of IBD are not well defined. In humans, the potential pathogenic processes involved in the development of IBD include persistent infections caused by environmental influences, enteric commensal bacteria, or reaction to antigens from foods, initiating acute and chronic intestinal inflammation. Consequently, this inflammation results in destruction of mucosal barriers as well as a dysregulation of the mucosal immune system. Bernstein et al. have surveyed the incidences of Crohn’s disease and ulcerative colitis in Canadian population [100]. The age group between 20 and 29 appears to show the highest incidences, with British Columbia showing the lowest (160/100,000) and Nova Scotia (318/100,000) showing the highest level of incidences. The disease affects children under age 10 to subjects over 80 years old and the incidences are much higher than anywhere else in the world. More than 200,000 people suffer from IBD in Canada, with an economic cost of >1.8 billion in direct and indirect costs (www.cdhf.ca). There are no identified preventive strategies or effective treatments for IBD, yet. The immunogenic mechanisms leading to IBD are complex, because the two types of IBD, UC and CD, show different immune responses to intestinal infections. As well, there is an increased risk for colon cancer development as a result of prolonged IBD.

The innate immune response at the gut mucosal interface is critical for human health, because it is the first line of defense against pathogen invasion and infection. An abnormal intestinal mucosal immune system can develop in patients who suffer from IBD and this can result in an inflammatory autoimmune response. There is a complex interplay between genetic susceptibility, commensal microbiota, intestinal epidermal cells, the immune system and the dietary and environmental factors that result in the development of IBD defining it as a multifactorial disease. Genetic studies, especially genome wide association studies have identified a number of risk-conferring loci that overlap both CD and UC, that implicate the role of IL 23, T helper (Th) 17 cells, autophagy, etc. [98]. The importance of dietary factors is highlighted by the observation that short chain fatty acids derived by microbial fermentation of dietary fiber bind to G-protein coupled receptor 43 (GPR43) and down regulate inflammation. This is again supported by studies involving *Gpr43* knockout mice, which show impaired inflammation protection [98]. Despite the fact that numerous novel therapeutic approaches are currently being developed for IBD, these approaches are based upon suppressing the immune responses by the use of drugs (steroids, non-steroidal anti-inflammatory drugs), or through using specific antibodies to block pro-inflammatory cytokines. However, excessive immune suppression may increase the risk of developing cancer [101]. Thus, immune suppression therapy may have a detrimental effect on patient health in the long term. One approach that could be taken to limit the damaging effects of many of the available drugs used in IBD treatment regimes is to combine these drugs with dietary intervention and/or nutraceutical supplementation, to enhance the beneficial effects of these drugs whilst reducing the amount of drugs required to provide a beneficial effect. A combination of conventional and alternative therapies has been shown to be a viable option in the treatment of IBD [102,103,104].

Nutritional and dietary interventions have recently become potential complimentary strategies for down regulating various inflammatory diseases [105]. Furthermore, recent research has shown that the patients with CD in Canada required micronutrient supplementation [106]. Polyphenols and carotenoids are outstanding candidates for amelioration of inflammatory diseases because of their potency as antioxidants and regulators of inflammatory immune responses. Dietary intake of foods containing polyphenols resulted in the down regulation of several inflammation markers in animal models and humans [107]. Consumption of fruit juices and products from grape and pomegranate at moderate level resulted in increased antioxidant function and the reduction of lipid peroxidation in the plasma [108,109]. The results from several studies show an inverse correlation between fruit and vegetable consumption and the expression of inflammation markers in blood, such as CRP (C-Reactive protein) and IL6 (interleukin 6) and several other inflammation markers. In a study involving 285 adolescent boys in the age range of 13–17 years, consumption of a fruit- and vegetable-rich diet was found to decrease the levels of inflammation markers such as CRP, IL-6, and TNF-α [110]. In a group of 120 men and women between the ages of 40–74, intake of polyphenol-rich blueberry extracts (300 mg/day for three weeks) caused a significant reduction in plasma levels of pro-inflammatory cytokines and chemokines (IL-4, IL-13, IL-8 and IFN-α) of the NF-κB pathway [111]. Similarly, increased consumption of sweet bing cherries (280 g/day) resulted in lowered levels of CRP and NO [112]. In a study involving elderly 70-year-old men, increased intake of food rich in antioxidants resulted in lowered cyclooxygenase, cytokine-mediated inflammation and oxidative stress [113]. Though several studies have been conducted on dietary polyphenol intervention using animal models [104], and a few studies in humans using curcumin intervention [114,115], there are no detailed reports on the effects, or utility of polyphenols for the management of IBD in humans.

### 7.1. Inflammatory Bowel Diseases and Antioxidative Capacity of Carotenoids to Reduce Oxidative Stress and Inflammation

Studies of colonic mucosal biopsies from patients either suffering from UC or CD have clearly shown that epithelial cells possess increased levels of inflammation marker compounds and decreased levels of anti-oxidant enzymes clearly suggesting the role of increased oxidative stress and decreased antioxidant defenses in cases of IBD [116]. However, persistent endogenous oxidative stress, such as that generated during chronic intestinal inflammation, often overwhelms the normal endogenous antioxidants [117]. Thus, dietary antioxidants are compelling candidates for use as nutraceuticals to enhance the function of the antioxidant defense system during inflammation [79,80,118]. It has already been shown that carotenoids have an enhancing effect on the immune system in vivo [119]. Consequently, Kawakami et al. identified a significant correlation between the serum oxygen radical scavenging capacity and β-carotene and retinol concentrations in UC patients [120]. In addition, carotenoid supplementation appears to be a potential nutritional intervention for patients suffering from IBD. Current research has validated that lycopene shows better chemopreventive activity than β-carotene in mitigating oxidative damage in tissue under UV exposure, but both chemicals contribute to reducing lipid peroxide levels [121]. Both β-carotene and lycopene have been identified to protect low-density lipoprotein (LDL) from oxidization [122,123]. LDL can be oxidized in vivo by myeloperoxidase (MPO), an intracellular enzyme secreted by macrophages and neutrophils, resulting in aggravating inflammation [124]. The MPO can act as an indicator of neutrophil infiltration at sites of damaged colon. Tran et al. detected an increasing activity of MPO in experimental IBD [125]. Even though a direct identification of oxidized-LDL (ox-LDL) in IBD has not been reported, CXCL16 (Chemokine (C-X-C motif)) ligand 16, a transmembrane protein functioning as a scavenger receptor for ox-LDL has been recently identified in the blood of both CD and UC patients [126]. Carotenoid intervention may thus reduce the levels of ox-LDL in IBD by influencing expression of CXCL16.

An increase in ROS has been identified in both UC and CD [19]. ROS contributes to redox imbalance of inflammatory autoimmune disease and inducing the intestinal epithelial lesions. As demonstrated in several studies, the dietary carotenoids can scavenge intracellular ROS at different steps of the pathway [127,128]. However, degradation of β-carotene can lead to the production of epoxides at the β-ionone ring and aldehydes with different chain lengths and these cleavage products are highly reactive and potentially toxic to cells [129]. Thereby, carotenoids without the β-ionone ring such as lycopene may be more promising as an exogenous antioxidant supplement. Moreover, the capacity of lycopene to quench radicals is more extensive than β-carotene [118]. Thus, lycopene, referred to as an optimal exogenous antioxidant, has a greater potential to ameliorate inflammatory diseases [122,130,131,132].

### 7.2. IBD and Immune-Modulating Activity of Carotenoids

Even though CD and UC result from complex genetic and environmental etiological influences, these diseases promote excessive immune responses and persistent inflammation in the intestinal epithelia and gut-associated lymphoid tissue (GALT). The main goal of regulating inflammatory immune responses in IBD is to restore the homeostasis in the mucosal immune system and the phagocytosis mediated by leukocytes. In pathological conditions of IBD, the inflammatory responses are mediated by a number of stress-associated kinase pathways including JNK/p38 MAPK and redox sensitive transcription factors NF-κB. The dysregulated activation of NF-κB via toll-like receptors (TLRs) may be a result of NOD2 ((Nucleotide-binding oligomerization domain-containing protein 2; synonymous to CARD 15-Caspase recruitment domain-containing protein 15; or inflammatory bowel disease protein 1 (IBD1)) gene mutation, which is highly correlated, with pathogenesis of IBD. NF-κB is able to motivate the production of pro-inflammatory signals such as NO by the activation of iNOS, and prostaglandins synthesized through the cyclooxygenase (COX) pathways, resulting in enhancing the severity of inflammation [101,133]. Generally, NF-κB plays a crucial role in regulating immune responses to infection. NF-κB up-regulates the expression of genes related to pro-inflammatory cytokines, enzymes and adhesion molecules as well as production of ROS in chronic inflammatory disease such as IBD [134]. The pro-inflammatory cytokine TNF-α, and oxidative stress generated during the inflammation phase, can in turn promote the activation of NF-κB. The activity of NF-κB involved in chronic inflammation of IBD has been analyzed by several studies. Schreiber et al. determined an increased level of NF-κB, p65, which is a subunit of the NF-κB complexes, in the lamina propria (LP) biopsy specimen from CD patients [135]. LP is a vital part of the gut-associated lymphoid tissue (GALT), which contains large members of T cells. Rogler et al. identified that the activation of NF-κB was significantly increased in the inflamed mucosa [136]. In another study, an enhanced NF-κB expression was found in inflamed mucosal biopsies of children suffering from CD [137]. Therefore, the suppression and regulation of NF-κB activation are promising approaches to modulate the IBD progression. The transcription factor NF-κB is very sensitive to oxidative stress (OS), ROS generated under the oxidative stress conditions might play a key role in modulating the dysregulation of immune responses in IBD. Carotenoids can quench ROS generated during the inflammation phase and potentially modulate the perpetuating stimulation of NF-κB pathway in IBD. Mannick et al. identified that β-carotene supplementation resulted in a significant reduction of iNOS in patients with *Helicobacter pylori* infection [138]. Bai et al. comprehensively analyzed the effects of β-carotene on the redox-based NF-κB activation in the lipopolysaccharide-stimulated macrophages. Their results showed that β-carotene inhibited iNOS and COX-2 expression, reduced the productions of pro-inflammatory cytokines TNF-γ and IL-1β, and suppressed NF-κB activation [139]. Thus, carotenoid intervention can potentially regulate the redox status of NF-κB activation in the IBD progression. However, the regulating effects of carotenoids on molecular mechanisms of NF-κB activation in IBD are not well defined. Further studies are required to analyze the influence of carotenoids on inflammatory gene expression during IBD progression.

## 8. Epigenetics and Cancer

Dietary compounds are the primary components that regulate gene expression by epigenetic mechanisms such as DNA methylation and histone modification through histone acetyl transferases (HATs) and histone deacetylases (HDACs). Long-term consumption of bioactive compounds may alter the epigenome and significantly contribute to the development of nutritional programs to prevent and treat metabolic diseases. Dietary bioactive compounds such as genistein, phenylisothiocyanate, curcumin, resveratrol, indole-3-carbinol, and epigallocatechin-3-gallate, all regulate HDAC and HAT activities, which may prevent cancer development [140,141]. Sulforaphane inhibits HDAC activity in a dose-dependent manner in colon cancer cells (HCT116) [142]. Sulforaphane induces acetylation of histones H3 and H4 in mouse tissues (ileum, colon, and prostate) and peripheral mononuclear cells [143]. Resveratrol, a polyphenol in grapes, blueberries, mulberries, cranberries, peanuts and red wine, is engaged in regulating signaling pathways involved in meiosis, cell growth, apoptosis, angiogenesis and tumor metastasis. Resveratrol effects are mediated via regulation of protein methylation and acetylation by targeting HDAC11, SIRT1, and HATp300 [144]. Curcumin inhibits HATp300 activity, leading to induction of cancer cell apoptosis via p53 and caspase [144].

Isoflavones are bioactives, which have been shown to demonstrate health benefits including cancer prevention. Soybean phytoestrogen genistein can induce post-translational changes in histones and increase the expression of tumor suppressor genes p21 (WAF1/CIP1) and P16 by regulating chromatin condensation via HAT expression in human prostate cancer cells. Genistein induced demethylation and SIRT1 inhibition-mediated acetylation of histone H3-K9 associated with the PTEN, CYCD, and FOXO3A promoters [145]. Polyphenols, including flavonoids, EGCG and green tea catechin, reduce the activity of Class I HDACs in prostate cancer cells [146]. Quercetin, a polyphenol found in apple, buckwheat and citrus, can activate SIRT1, and NAD-dependent deacetylase. Quercetin is able to inhibit the expression of TNF-induced IFN-γ- inducible protein 10 (IP-10) and macrophage inflammatory protein 2 (MIP-2). Consequently, It blocks post-translational modifications (acetylation and phosphorylation) of H3 histones through p300/CBP induction. As a result, the promoters of pro-inflammatory genes associated with H3 histones get affected by inhibiting cofactor recruitment at the chromatin [147]. Moreover, through inhibition of HDAC and DNMT1, quercetin inhibits the cell cycle and induces apoptosis consequently, suppressing tumor growth and angiogenesis [148].

Glucosinolates such as glucobrassicin, in *Brassica* vegetables such as broccoli, brussels sprouts, cabbage and kale, can produce diindolylmethane (DIM) and indol-3-carbinol (I3C). The exposure to DIM has shown a significant reduction in the levels of Class I HDACs, associated with the increase in histone acetylation of the promoters of cell cycle kinase inhibitors p21WAF1 and p27, which halt the cell cycle and increase DNA damage in colon cancer cells [149]. Organosulfur compounds diallyl disulfide (DADS) and its active metabolite S-allyl mercaptocysteine (SAMC) in allium vegetables such as garlic, induced an increase in histone H3K14 acetylation associated with the activation of p21 promoter and inhibition of the proliferation of breast and colon cancer cells [150].

## 9. MicroRNA, Nutrition and Cancer

MicroRNA (miRNA) are small noncoding RNA molecules (about 22 nucleotides in length) involved in RNA silencing and post-transcriptional regulation of gene expression [151]. They negatively regulate gene expression by pairing with 3’-untranslated regions of target mRNAs, inducing deadenylation and translational repression in a cell-type specific manner. MicroRNAs genes are transcribed from classical genomic intron and exon regions. Their primary transcripts process by successive actions of a nuclear (Drosha) and a cytoplasmic (Dicer) RNAase III. miRNAs became particularly attractive in oncology since they are simple, stable molecules easy to detect in tissues and blood circulation. Increasing evidence suggests that miRNAs are involved in broad genomic processes including the regulation of expression of oncogenic and tumor-suppressive genes [152,153,154]. Studies have shown different miRNA profiles in tumor tissues compared to normal tissues [155]. Importantly, specific miRNA profiles seem to be present in different types of cancer [156]. Phytochemicals that regulate expression and action of miRNA during cancer development including apoptosis, cell cycle regulation, differentiation, inflammation, angiogenesis and metastasis may have a potential to consider as a candidate for cancer therapy [157]. However, specific targeting and bioavailability of phytochemicals need to be better understood before developing them into pharmaceuticals.

miRNA acts as tumor suppressors or oncogenes [158]. They are capable to influence cancer in multiple ways like sustaining proliferative signaling, regulating the genomic stability and metabolisms of cancerous cells, mediating the immune responses in cancer, evading growth suppressors, resisting cell death, enabling replicative immortality, inducing angiogenesis, and activating invasion and metastasis [159,160].

Phytochemicals can regulate the expression of various miRNAs in different types of cancers [157]. EGCG treatment of HepG2 liver cancer cells caused a significant change in 13 miRNA including miR-16 up-regulation and 48 miRNA down-regulation. EGCG treatment raised the level of miR-16, leading to a decrease in Bcl2 level and induction of apoptosis [161]. Curcumin has also shown the potential to increase 11 and decrease 18 miRNA expression following 72 h of incubation in human pancreatic cancer cell cultures. Curcumin up-regulated miR-22 by suppressing the expression of its targets, Sp1 and ERRα1 which are transcription factors [162]. Curcumin also enhanced the expression of miR-15a and miR-16 in MCF-7 breast cancer cells, leading to apoptosis [163]. Curcumin also reduced miR-21 promoter activity and expression in primary colon cancer [164].

Resveratrol is able to decrease the expression of oncogenic miRNAs in human colon cancer cells, such as miR-17, miR-21, miR-25, miR-92a-2, miR-103-1, and miR-103-2, and restore tumor suppressor miR-663 [165]. In pancreatic cancer cells, resveratrol inhibited the oncogenic miR-21 [166], and prevented cell growth and induction of apoptosis by increasing miR-34a in colon cancer cells [167]. Table 1 summarizes some examples in terms of effects of phytochemicals on different types of cancers miRNA.

## 10. Metabolic Stability of Plant Bioactives

Metabolic stability of anthocynins during their transit through gastrointestinal tract is another interesting aspect that may influence the bioaccessibility, bioavailability and the beneficial effects of bioactives such as polyphenols. Anthocyanins are unstable at alkaline pH, and undergo ring fission during their transit in small intestine. Further, the components that escape the intestinal conditions are subjected to colonic fermentation; generating a variety of simple components such as phenolic acids. It is believed that, these phenolic acids have a beneficial role in providing health benefits, probably through their antioxidant function, or through modulation of antioxidant systems. Structural changes of anthocyanins during intestinal digestion and colonic fermentation by microbiome influence their absorption. Accordingly, in vitro research on blueberry polyphenols showed that stability of the anthocyanins depends on the nature and number of sugars attached to the benzopyran ring and the variety of acidic components (e.g., acetoyl, malonoyl, caffeoyl, and coumaroyl groups) that are linked to sugar moiety. Such changes in phenolic compounds may enhance their function by reducing the risk of developing chronic diseases such as cancer through multiple mode of action [176,177].

## 11. Conclusions

**Figure 2:**
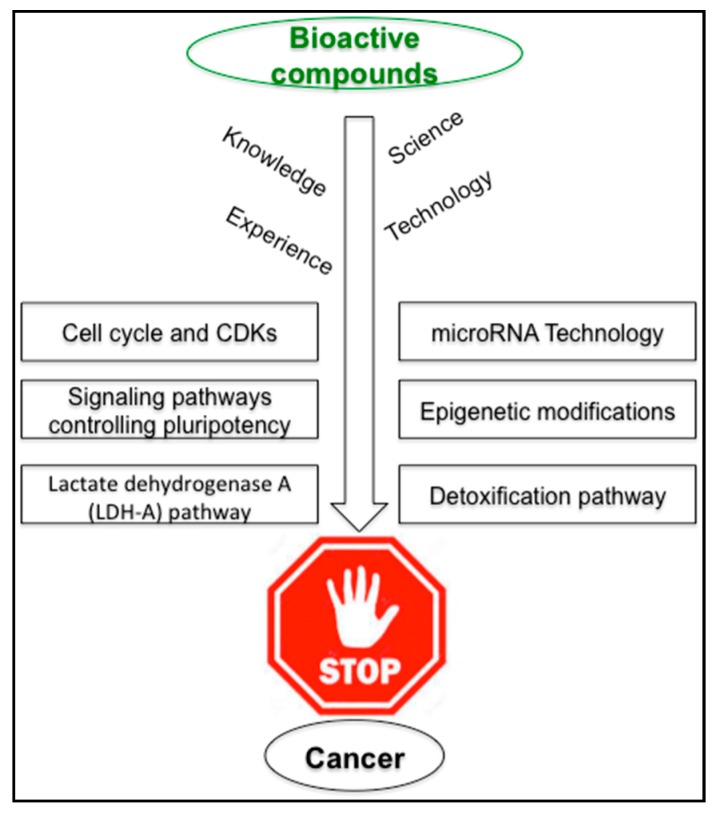
The mechanisms used by bioactive nutrients to reduce cancer risk.

As a result of new approaches, the concept of achieving ideal health is changing, and focusses on the importance of a healthy lifestyle centered on diet and exercise. Diet plays a crucial role in the regulation of metabolic pathways genetically and epigenetically. Many epidemiologic studies have shown positive influences of fruit- and vegetable- enriched diets in preventing chronic diseases such as cancer. Although numerous bioactive compounds appear to have beneficial effects in preventing cancers, strong scientific evidence, based on clinical studies, needs to be gathered before offering science-based dietary recommendations. By the help of modern genetics, chemistry and molecular biology, nutrition research will increasingly be able to apply new discoveries to develop designer functional foods by adding specific bioactive characteristics for preventing and reducing the risk of cancer development. Herbal medicines have been used since ancient times. They are usually a mixture of several compounds, which can affect cells, but whether it is an impact of a single compound or a specific combination, is poorly understood. It is time to connect all these knowledge and experiences gathered over thousands of years in several civilizations to technology. Thus, functional components of food can be effectively applied in the treatment and prevention of cancer [178,179]. Figure 2 depicts how science, knowledge, experience and technology can be rationally applied in understanding the regulation of signaling pathways such as those controlling pluripotency, LDH-A pathway, epigenetic modifications, detoxification pathway and miRNA action to combat cancer.

## Figures and Tables

**Table 1:** Effects of phytochemicals on different types of cancer miRNA.

Phytochemicals	miRNA	Cancer	References
Curcumin	miR-22, miR-15a, miR-16, miR-21	Pancreatic cancer, Breast cancer, Colon cancer	[162,163,164]
Diindolylmethane (DIM)	miR-200, let-7, miR-21	Pancreatic cancer, Breast cancer	[168,169]
EGCG	miR-98-5p, miR-13, miR-48, miR-16, miR-21	Lung cancer, HCC, Prostate cancer, Pancreatic cancer	[161,167,170,171]
Genistein	miR-221, miR-222, miR-27a	Prostate cancer, Ovarian cancer	[172,173]
Quercetin	miR-27a, Let-7	Colorectal cancer, Pancreatic cancer	[174,175]
Resveratrol	miR-17, miR-21, miR-25, miR-92a-2, miR-103-1 and miR-103-2, miR-663, miR-34a	Colon cancer, Pancreatic cancer	[165,166,167]

## Abbreviations

NF-κB	Nuclear factor kappa B
IBD	Inflammatory bowel diseases
STAT3	Signal transducer and activator of transcription 3
MCF-7	Michigan cancer foundation-7
MCF-10A	Michigan cancer foundation-10A
LEF1	Lymphoid enhancer-binding factor 1
PTGS2	Prostaglandin-endoperoxide synthase 2 (prostaglandin G/H synthase and cyclooxygenase)
PRKCE	Protein kinase C epsilon
ROS	Reactive oxygen species
TNF-α	Tumor necrosis factor alpha
Ap-1	Activator protein-1
CDKs	Cyclin-dependent kinases
PEITC	Phenethyl isothiocyanate
ATM	Ataxia telangiectasia mutated
ChK1	Checkpoint kinase 1
AR	Androgen receptor
MMPs	Matrix metalloproteinases (MMP-2 and MMP-9)
CK1α	Casein kinase-1a
SK3 β	Glycogen synthase kinase-3 β
LDH-A	Lactate dehydrogenase A LDH-A
HIF	Hypoxia inducible factor
PDH	Pyruvate dehydrogenase complex
GST	Glutathione *S*-transferase
UDP-GT	UDP-glucuronyl transferase
Nrf2	Nuclear factor F-related factor 2
Keap1	Kelch-like ECH-associated protein 1
ARE	Antioxidant responsive element
Keap1/Nrf2/ARE	Kelch ECH associating protein 1/NF-E2-related factor 2/Antioxidant Response Elements
I3C	Indole 3-carbinol
CD	Crohn’s disease
UC	Ulcerative colitis
GPR43	G-protein coupled receptor 43
CRP	C-Reactive protein
ILs	Interleukins
LDL	Low-density lipoprotein
MPO	Myeloperoxidase
ox-LDL	Oxidized-LDL
CXCL16	Chemokine (C-X-C motif) ligand 16-a
IFNs	Interferons
GALT	Gut-associated lymphoid tissue
TLRs	Toll-like receptors
NOD2	Nucleotide-binding oligomerization domain-containing protein 2
COX	Cyclooxygenase
LP	Lamina propria
OS	Oxidative stress
HATs	Histone acetyl transferases
HDACs	Histone deacetylases
IP-10	Inducible protein 10
MIP-2	Macrophage inflammatory protein 2
DIM	Diindolylmethane
DADS	Diallyl disulfide
SAMC	*S*-allyl mercaptocysteine
